# The Mechanism behind Bacterial Lipoprotein Release: Phenol-Soluble Modulins Mediate Toll-Like Receptor 2 Activation via Extracellular Vesicle Release from Staphylococcus aureus

**DOI:** 10.1128/mBio.01851-18

**Published:** 2018-11-20

**Authors:** Katja Schlatterer, Christian Beck, Dennis Hanzelmann, Marco Lebtig, Birgit Fehrenbacher, Martin Schaller, Patrick Ebner, Mulugeta Nega, Michael Otto, Dorothee Kretschmer, Andreas Peschel

**Affiliations:** a Department of Infection Biology, Interfaculty Institute for Microbiology and Infection Medicine Tübingen (IMIT), University of Tübingen, Tübingen, Germany; b Electron-Microscopy, Department of Dermatology, University Hospital Tübingen, Tübingen, Germany; c Department of Microbial Genetics, Interfaculty Institute for Microbiology and Infection Medicine Tübingen (IMIT), University of Tübingen, Tübingen, Germany; d Pathogen Molecular Genetics Section, Laboratory of Bacteriology, National Institute of Allergy and Infectious Diseases, National Institutes of Health, Bethesda, Maryland, USA; Institut Pasteur; Institut Pasteur

**Keywords:** *Staphylococcus aureus*, leukocytes, lipoproteins, pore-forming toxins, Toll-like receptors, vesicles

## Abstract

The innate immune system uses Toll-like receptor (TLR) 2 to detect conserved bacterial lipoproteins of invading pathogens. The lipid anchor attaches lipoproteins to the cytoplasmic membrane and prevents their release from the bacterial cell envelope. How bacteria release lipoproteins and how these molecules reach TLR2 remain unknown. Staphylococcus aureus has been described to liberate membrane vesicles. The composition, mode of release, and relevance for microbe-host interaction of such membrane vesicles have remained ambiguous. We recently reported that S. aureus can release lipoproteins only when surfactant-like small peptides, the phenol-soluble modulins (PSMs), are expressed. Here we demonstrate that PSM peptides promote the release of membrane vesicles from the cytoplasmic membrane of S. aureus via an increase in membrane fluidity, and we provide evidence that the bacterial turgor is the driving force for vesicle budding under hypotonic osmotic conditions. Intriguingly, the majority of lipoproteins are released by S. aureus as components of membrane vesicles, and this process depends on surfactant-like molecules such as PSMs. Vesicle disruption at high detergent concentrations promotes the capacity of lipoproteins to activate TLR2. These results reveal that vesicle release by bacterium-derived surfactants is required for TLR2-mediated inflammation.

## Abstract (precis)

Our study highlights the roles of surfactant-like molecules in bacterial inflammation with important implications for the prevention and therapy of inflammatory disorders. It describes a potential pathway for the transfer of hydrophobic bacterial lipoproteins, the major TLR2 agonists, from the cytoplasmic membrane of Gram-positive bacteria to the TLR2 receptor at the surface of host cells. Moreover, our study reveals a molecular mechanism that explains how cytoplasmic and membrane-embedded bacterial proteins can be released by bacterial cells without using any of the typical protein secretion routes, thereby contributing to our understanding of the processes used by bacteria to communicate with host organisms and the environment.

## INTRODUCTION

The innate immune system uses pattern recognition receptors (PRRs) such as the Toll-like receptors (TLRs) to detect conserved microbe-associated molecular pattern molecules (MAMPs) as a hallmark for the presence of invading pathogens (1). TLR2 is the major mammalian PRR that senses the presence of Staphylococcus aureus, one of the most frequent and aggressive bacterial causes of wound, soft tissue, lung, and bloodstream infections (2). TLR2 senses bacterial lipoproteins, the characteristic lipid anchor of which is absent from human molecules. S. aureus uses a large panel of lipoproteins, most of which are components of ATP-binding cassette (ABC) import systems (3). The lipid anchor attaches lipoproteins to the outer surface of the cytoplasmic membrane, which ensures an appropriate localization in the bacterial cell envelope but also prevents their release and detection by TLR2. How bacteria release lipoproteins and how they reach TLR2 have remained incompletely understood.

We recently reported that S. aureus releases substantial amounts of lipoproteins into culture supernatants only when surfactant-like small peptides, the phenol-soluble modulins (PSMs), are strongly expressed (4). PSMs have direct proinflammatory and leukocyte-recruiting activity through activation of the human and mouse formyl-peptide receptor (FPR) 2, a G-protein-coupled receptor (5, 6). Moreover, PSMs can modulate host membrane functions, including the cytolysis of human cells, at high concentrations (7, 8). Therefore, PSMs are among the most critical and aggressive S. aureus virulence factors. S. aureus produces seven to eight different PSMs, including the short α-type PSMs (PSMα1 to -4 and the δ-toxin) and the twice-as-long β-type PSMs (PSMβ1 and -2) (9). An additional α-type PSM, PSMmec, is encoded on the mobile genetic element SCCmec type II and III of some methicillin-resistant S. aureus (MRSA) strains (10). Interestingly, PSMα1-4 and δ-toxin are abundant on the S. aureus cell surface (11). This feature demonstrates that PSMs interact not only with the eukaryotic cell envelope but also with the PSM producer’s own membrane. We have also previously shown that PSMs mobilize lipoproteins from the cytoplasmic membrane of S. aureus (4), but the precise mechanism has remained unclear.

TLR2 activation by S. aureus lipoproteins can contribute to massive inflammation (4) but can also elicit anti-inflammatory responses (12) in a context-dependent, only partially elucidated way. TLR2-deficient mice are more susceptible to death from systemic S. aureus infections (4), and S. aureus mutants without lipoproteins have abrogated virulence (13). However, S. aureus can modulate the release and activity of lipoproteins through several mechanisms. While many commensal bacteria produce highly active lipoproteins, S. aureus and the opportunistic pathogen Staphylococcus epidermidis incorporate a third long-chain fatty acid into their lipoproteins, which reduces the TLR2-stimulating capacity of lipoproteins (14). Many S. aureus strains produce SSL3, a specific inhibitor of TLR2 (15). Moreover, the release of lipoproteins is controlled by the quorum-sensing Agr regulation system, which modulates the expression of lipoprotein-releasing PSM peptides (4).

S. aureus has recently been found to release membrane vesicles (MVs), which can stimulate TLR2 and contribute to inflammation, for instance, in the skin (16–18). However, it is unclear whether such MVs contain a relevant percentage of S. aureus lipoproteins, and the molecular mechanisms responsible for vesicle release remain unknown.

We demonstrate here that PSM peptides promote the release of MVs from the cytoplasmic membrane of S. aureus by increasing membrane fluidity, and we provide evidence that bacterial turgor is the driving force for vesicle budding under hypotonic osmotic conditions. Most of the lipoproteins released by S. aureus are embedded in MVs, which, when disrupted by high detergent concentrations, show higher capacity to activate TLR2.

## RESULTS

### Lipoproteins and PSMs released by S. aureus are associated with high-molecular-weight aggregates.

PSMs might release lipoproteins from the cytoplasmic membrane of S. aureus either as individual molecules with hydrophobic fatty acid chains that are shielded by amphipathic PSM peptides or embedded into larger aggregates, which may be kept together by hydrophobic interactions. To discriminate between these two possibilities, the culture filtrates of S. aureus USA300 wild type, which contain large amounts of lipoproteins, and of the isogenic PSM mutant, which releases only residual amounts of lipoproteins, were size-fractioned using centrifugal concentrator cartridges with a molecular weight cutoff of 100 kDa. S. aureus lipoproteins and PSM peptides have masses of 33 to 37 (19) and 2.2 kDa (7), respectively, and would be found in the <100-kDa fraction if they were not associated with larger aggregates.

**FIG 1:**
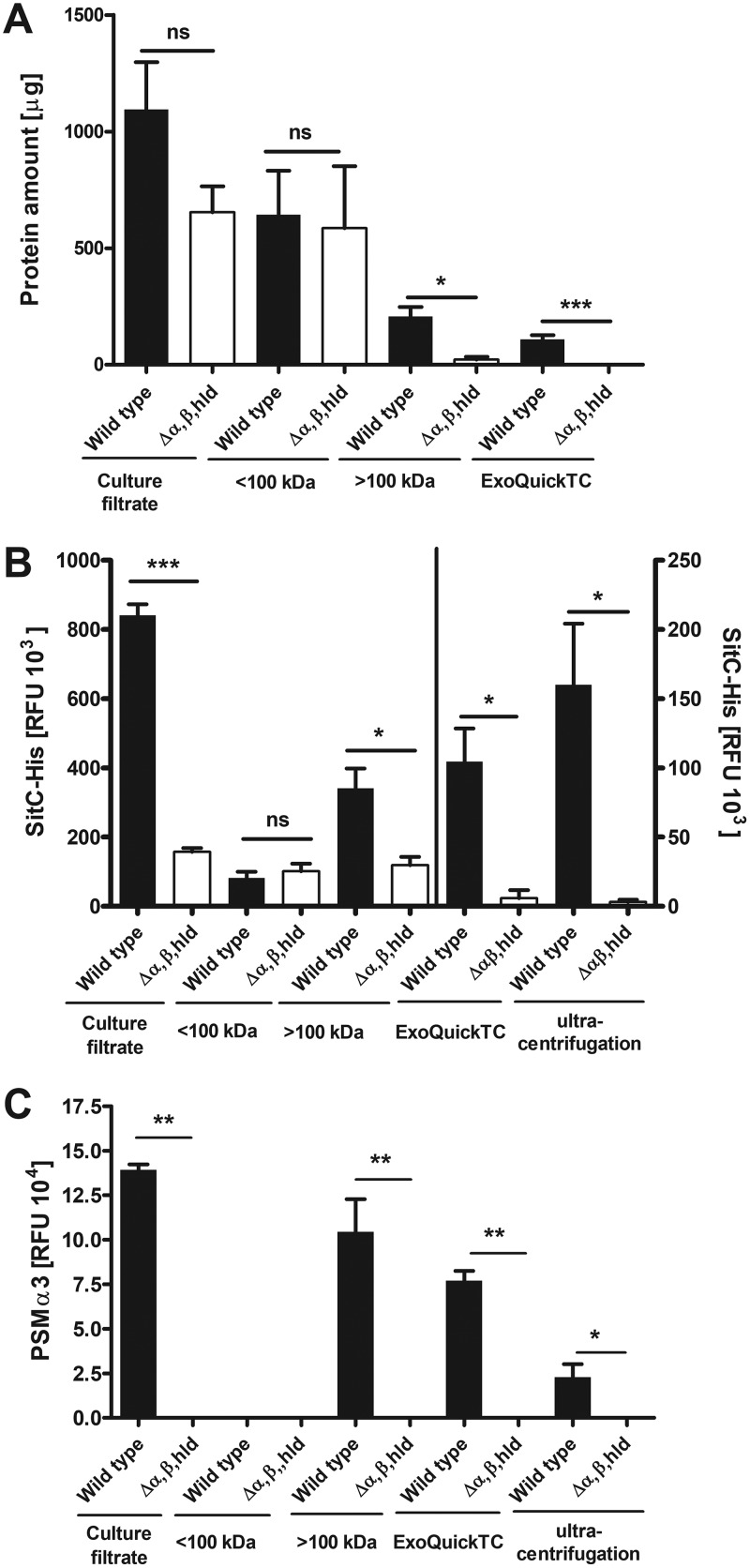
Detection of proteins, SitC, and PSMα3 in different wild-type and PSM mutant (Δ*α,β,hld*) fractions. Culture filtrates were fractioned with 100-kDa centrifugal concentrator cartridges. Fractions were analyzed for protein amounts (A) and amounts of both the model lipoprotein SitC (B) and PSMα3 (C). Culture filtrates, low-molecular-weight (<100-kDa), and high-molecular-weight (>100-kDa) fractions as well as membrane vesicles (MVs) isolated by gradient ultracentrifugation and ExoQuickTC were analyzed. Data represent means ± SEMs from at least three independent experiments. ns, not significant; *, *P* < 0.05; **, *P* < 0.01; ***, *P* < 0.001, significant difference versus USA300 wild type as calculated by the unpaired, two-tailed Student *t* test.

Most of the proteins in S. aureus culture filtrates were found in the flowthrough (<100-kDa) fraction, indicating that the majority of secretory proteins do not form larger aggregates (Fig. 1A). However, the PSM mutant contains approximately 15-fold less protein in the high-molecular-weight (>100-kDa) fraction than the wild type (Fig. 1A), indicating that S. aureus releases proteins embedded in larger aggregates in a PSM-dependent fashion.

When the size-fractioned culture filtrates from S. aureus were analyzed for amounts of the model lipoprotein SitC (18, 19), using a USA300 strain expressing SitC with a C-terminally linked His tag (SitC-His), most SitC was detected in the high-molecular-weight fraction of the wild type, whereas all fractions of the PSM mutant contained only small amounts of SitC (Fig. 1B). This finding is in agreement with our previous report on the essential role of PSMs for lipoprotein release (4) and indicates that most SitC is enclosed in high-molecular-weight aggregates. Furthermore, the amount of PSMα3 in the different fractions was analyzed by immunoblotting, and PSMα3 was also detected mostly in the >100-kDa fraction of the wild type (Fig. 1C). This finding indicates that PSMs do not only mobilize lipoproteins but also remain associated with them in large aggregates.

### S. aureus-released lipoproteins are components of membrane vesicles.

**FIG 2:**
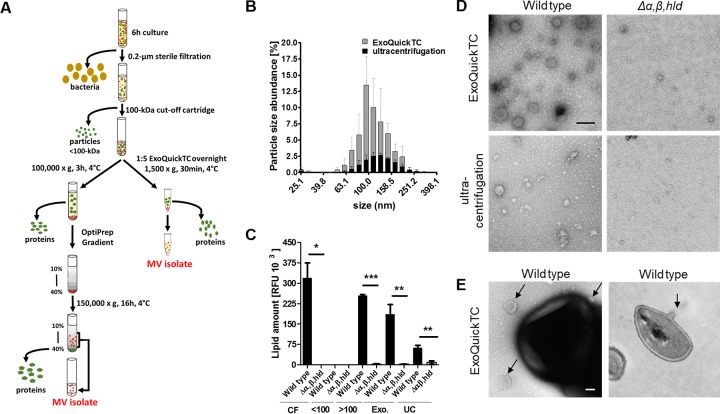
S. aureus MV biogenesis is PSM dependent regardless of the MV isolation method. (A) Schematic representation of the vesicle isolation using the ExoQuickTC kit and gradient ultracentrifugation. (B) Particle size analysis via dynamic light scattering in wild-type MVs isolated by ExoQuickTC and gradient ultracentrifugation. (C) Lipid amount (FM4-64 dye) in culture filtrates (CF) and low-molecular-weight (<100-kDa), high-molecular-weight (>100-kDa), and MV fractions isolated via ExoQuickTC (Exo.) and gradient ultracentrifugation (UC). (D) Electron microscopic (TEM) images of wild-type and Δ*α,β,hld* MVs after isolation with the ExoQuickTC isolation kit and OptiPrep gradient ultracentrifugation (scale bar, 0.1 μm). (E) TEM images of USA300 wild-type bacteria and associated membrane vesicles (indicated by black arrows) in a negative-stained and an ultrathin section (scale bar, 0.1 μm). Data in panel B represent means and data in panel C represent means ± SEMs from at least three independent experiments. *, *P* < 0.05; **, *P* < 0.01; ***, *P* < 0.001, significantly different versus USA300 wild type, as calculated by the unpaired, two-tailed Student *t* test. Data in panels D and E show one representative example.

Since the aggregates containing S. aureus lipoproteins were found to be over 100 kDa in size, it is possible that these aggregates are large hydrophobic structures like membrane vesicles (MVs), which were previously reported to show a size range of 20 to 130 nm (16). To analyze if the S. aureus lipoproteins in culture supernatants are indeed embedded in MVs, the high-molecular-weight fractions of S. aureus USA300 and the isogenic PSM mutant (Δα,β,*hld*) were additionally enriched for MVs by MV-precipitation reagent (ExoQuickTC) or density gradient ultracentrifugation (OptiPrep) (Fig. 2A), which have been reported to facilitate the isolation of MVs. The wild-type fraction, which was enriched via ExoQuickTC, contained approximately 50% of the protein amount found in the high-molecular-weight fraction, while no proteins could be found in the corresponding fraction from the PSM mutant (Fig. 1A). Fractions isolated by density gradient ultracentrifugation also showed decreased protein amounts in the PSM mutant fraction compared to the wild type (see Fig. S1A in the supplemental material).

10.1128/mBio.01851-18.1 FIG S1 Determination of protein amounts, MV size, and cargo in MV isolates of wild-type and Δ*α,β,hld* strains. (A) Silver staining of MV isolates from USA300 pTX SitC-His and Δ*α,β,hld* pTX SitC-His recovered via OptiPrep gradient ultracentrifugation. Fractions showing similar protein patterns (35 to 20% OptiPrep) were pooled and referred to as MV isolates. (B) Particle size analysis via dynamic light scattering in wild-type and PSM mutant MVs isolated by ExoQuickTC. (C) Flow cytometric analysis of GFP-positive particles additionally positive for lipids (FM4-64) after isolation from USA300 carrying the pTX143-S3-GFP plasmid. Data in panels B and C represent means from at least three independent experiments. Data in panel A show one representative of at least three independent experiments. Download FIG S1, TIF file, 0.8 MB. Copyright © 2018 Schlatterer et al. 2018 Schlatterer et al. This content is distributed under the terms of the Creative Commons Attribution 4.0 International license.

The particle sizes in the MV-containing fractions gained by both isolation methods were determined by dynamic light scattering analysis and were found to be in the range of 60 to 200 nm with a maximum around 100 nm (Fig. 2B and Fig. S1B), which is similar to the reported sizes of S. aureus MVs. Furthermore, by using transmission electron microscopy (TEM), we observed vesicles with average diameters of 80 to 100 nm in wild type but not in PSM mutant fractions prepared via OptiPrep or ExoQuickTC (Fig. 2D). Moreover, individual S. aureus cells were found to constrict MV-like structures (Fig. 2E).

**FIG 3:**
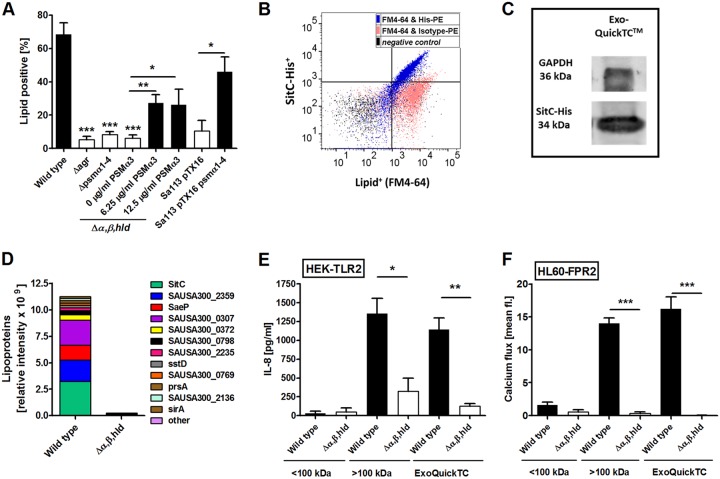
Composition and host-activating capacity of MVs isolated from wild-type and Δ*α,β,hld* bacterial cultures. (A) Flow cytometry analysis of MVs recovered from strains without PSM expression such as USA300 Δ*agr*, USA300 Δ*psmα1-4*, the PSM mutant (*Δα,β,hld;* lacking all PSMs), or the *agr*-deficient laboratory strain SA113 revealed substantially reduced MV amounts. Addition of synthetic PSMα3 to the PSM mutant culture, or complementation of SA113 with a plasmid carrying *psmα1-4*, successfully restored the release of MVs. (B) Lipid membrane (FM4-64^+^)-positive particles from USA300 Δ*spa* pTX SitC-His are also SitC-His positive when analyzed by flow cytometry. (C) Immunoblotting of wild-type ExoQuickTC-isolated MVs detecting the cytoplasmic protein glyceraldehyde-3-phosphate dehydrogenase (GAPDH) and the model lipoprotein SitC. (D) Proteomic analysis reveals the presence of other lipoproteins in addition to SitC in wild-type ExoQuickTC-isolated MVs. (E) Larger-than-100-kDa and MV fractions from wild type can activate TLR2-transfected HEK293 cells, resulting in the secretion of IL-8 cytokines. All <100-kDa fractions as well as all Δ*α,β,hld* fractions fail to induce a strong IL-8 secretion. (F) Wild-type MV and high-molecular-weight (>100-kDa) fractions induce calcium influx in FPR2-transfected HL60 cells. All other fractions fail to induce calcium influx. Data in panels A, E, and F represent means ± SEM from at least three independent experiments. *, *P < *0.05; **, *P < *0.01; ***, *P < *0.001, significant difference versus USA300 wild type, as calculated by the unpaired (A) or paired (E and F) two-tailed Student *t* test. Data in panel D represent means of three independent experiments, and data in panels B and C are each representative of three independent experiments.

The presence of membrane lipids is a hallmark for MVs. Although microscopically, more MVs are visible in wild-type than in PSM mutant MV preparations, it is difficult to quantify vesicles by TEM. We used a specific fluorescent membrane dye (FM4-64) to quantify the amount of lipids in the MV and size-separated fractions. All fractions containing MVs exhibit substantial FM4-64 signals. Moreover, the significantly decreased amounts of lipids in all fractions of the PSM mutant compared with the wild type (Fig. 2C) match the results from protein detection and TEM analysis. Additionally, MVs could be analyzed by flow cytometry upon staining with FM4-64 in wild-type ExoQuickTC vesicle isolates but not in isolates from the PSM mutant (*Δα,β,hld*) or a mutant lacking only PSMα1 to -4 (Δ*psm*α*1-4*) (Fig. 3A). Likewise, in USA300 Δ*agr* and the *agr*-deficient laboratory strain SA113, which do not express PSMs, MV release was substantially reduced (Fig. 3A). Addition of synthetic PSMα3 to the PSM mutant culture, or complementation of SA113 with a plasmid carrying *psmα1-4*, successfully restored the release of MVs (Fig. 3A), confirming the PSMα1 to -4-dependent MV biogenesis in S. aureus. Altogether, this corroborates recent findings on the PSM-promoted release of proteins, lipids, nucleic acids, and ATP from S. aureus cells (16).

To analyze the presence of SitC and PSMs in S. aureus-released MVs, the vesicle preparations were also subjected to immunoblotting. Indeed, SitC and PSMα3 were found in the ExoQuickTC- or OptiPrep-isolated MV fractions of S. aureus wild type but not of the PSM mutant (Fig. 1B and C and 3D), which confirms that SitC and PSMs do not occur in culture filtrates as individual molecules but as a components of MVs. Using flow cytometry, colocalization of lipid membranes (FM4-64) and SitC (Fig. 3B) could be confirmed using a His-tag-specific antibody to detect SitC-His. Since MVs are constricted from the cytoplasmic membrane, they are likely to contain cytoplasmic proteins. In addition to the lipoprotein SitC, the plasmid-expressed cytoplasmic green fluorescent protein (GFP) was also found to colocalize with lipids (Fig. S1C) when analyzed by flow cytometry. Immunoblotting of wild-type MV fractions additionally confirm an association of the cytoplasmic protein glycerophosphate dehydrogenase (GAPDH) with MVs (Fig. 3C). MV preparations were furthermore subjected to proteomic analysis, and the wild-type MVs were found to contain cytoplasmic proteins and other lipoproteins in addition to SitC (Fig. 3D and Data Set S1).

10.1128/mBio.01851-18.5 DATA SET S1 Quantitative label-free proteomic analysis of MVs isolated by ExoQuickTC. Three biological replicates of USA300 wild-type and PSM mutant MVs isolated by ExoQuickTC were analyzed for protein content and composition. Label-free quantification (LFQ) protein intensities are listed for detected proteins in the MV isolates (sheet: quantitative label-free). Proteins were sorted for their subcellular localization. Mean LFQs of each protein were compared between wild type and PSM mutant (sheets: cytoplasmic, cytoplasmic membrane, cell wall, extracellular, unknown and lipopeptides). Download Data Set S1, XLSX file, 0.1 MB. Copyright © 2018 Schlatterer et al. 2018 Schlatterer et al. This content is distributed under the terms of the Creative Commons Attribution 4.0 International license.

The high-molecular-weight and the MV-enriched fractions were also tested for their TLR2- and FPR2-activating capacities using TLR2-transfected HEK293 cells and FPR2-transfected HL60 cells, respectively. In agreement with the presence of large amounts of lipoproteins and PSMs in high-molecular-weight and MV fractions from the USA300 wild type (Fig. 1B and C), high TLR2- and FPR2-stimulating activities were observed in these fractions (Fig. 3E and F). In contrast, the same volumes of the MV fractions from the PSM mutant were largely inactive, which is in agreement with the low MV content. Likewise, all analyzed fractions <100 kDa showed only minimal TLR2 or FPR2 activity (Fig. 3E and F). Thus, the vast majority of TLR2-activating lipoproteins and FPR2-activating PSMs in S. aureus culture supernatants did not occur as individual molecules but as components of MVs. Altogether, these data confirm that lipoproteins and cytoplasmic proteins in the S. aureus culture supernatant are enclosed in MVs.

### PSMα3 increases S. aureus membrane fluidity and promotes turgor-dependent MV budding.

**FIG 4:**
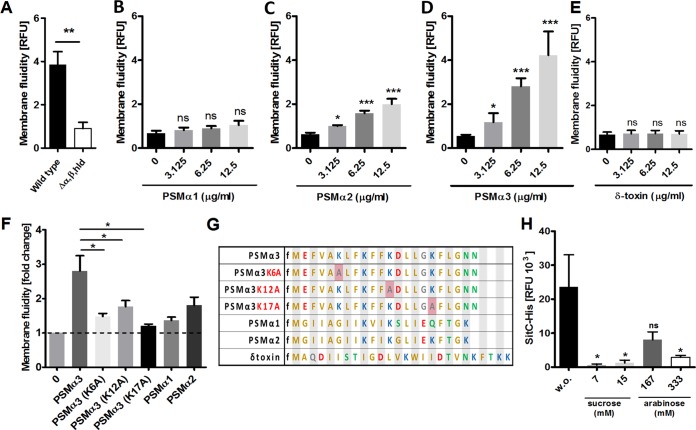
PSMα3 increases membrane fluidity, while turgor pressure influences vesicle formation. (A) Measurements of membrane fluidity in USA300 wild-type and USA300 Δ*α,β,hld* bacteria containing 10% autologous culture filtrate reveal higher intrinsic fluidity of the wild-type cytoplasmic membrane. (B to E) Membrane fluidity of USA300 Δ*α,β,hld* bacteria after addition of the indicated synthetic PSMs. (F) Amino acid exchanges in the sequence of PSMα3 strongly impair the positive effect of the native PSMα3 on the membrane fluidity. Dashed line represents a fold change of one (base value). (G) Sequences of the synthetic PSMs and the PSMα3 alanine substitution variants. (H) Immunoblot analysis of MV-bound SitC released from USA300 pTX SitC-His after addition of the indicated concentrations of sucrose or arabinose or without (w.o.) addition to the bacterial culture. (A to F) Membrane fluidity was measured as relative fluorescence units (RFU) and calculated as ratio of excimer/monomer RFU of the lipophilic pyrene probe. Data in panels A to F and H represent means ± SEMs from at least three independent experiments; ns, not significant; *, *P* < 0.05; **, *P* < 0.01; ***, *P < *0.001, significant difference versus untreated bacteria as calculated by the unpaired, two-tailed Student *t* tests.

While eukaryotic cells use sophisticated molecular machines to constrict MVs, no such systems are known in prokaryotes (17). Because PSMs have surfactant-like properties (9), it is tempting to assume that they alter the properties of the S. aureus cytoplasmic membrane in a way that favors the spontaneous budding of MVs. A membrane fluidity assay based on the fluorescence of a membrane-integrating fluorescent dye (20) was used to analyze the impact of PSMs on S. aureus membrane properties. S. aureus wild type exhibited a higher intrinsic membrane fluidity than the PSM mutant (Fig. 4A), suggesting that PSMs may increase fluidity. In accordance with this idea, the addition of synthetic PSMs to PSM mutant bacteria also led to increased membrane fluidity. The PSMα peptides, in particular PSMα3 and -α2, had a much stronger impact on membrane fluidity than δ-toxin (Hld) (Fig. 4B to E), which is in agreement with the documented, particularly high capacity of PSMα3 to disrupt membranes (7). The lytic effect of PSMs, especially PSMα3, is most likely a result of their strong α-helical and amphipathic structure (7). Analysis of an alanine substitution library of PSMα3 has revealed the importance of the positively charged lysine residues for the lytic capacity of PSMα3 (21). Selected alanine substitution variants of PSMα3 showed an impaired ability to increase the membrane fluidity in comparison to PSMα3, indicating that the amphipathic, α-helical structure is important for the increase in membrane fluidity (Fig. 4F and G).

While increased membrane fluidity can promote vesicle budding (22), the mechanism providing the driving force for this energy-dependent process has remained unknown. S. aureus usually encounters hypotonic conditions in human body fluids or in culture media, and it is tempting to assume that turgor pressure may be the driving force for vesicle constriction. To evaluate this hypothesis, S. aureus was incubated in the presence of increasing concentrations of the solutes sucrose and arabinose, which do not affect S. aureus growth (Fig. S2A) but are thought to decrease the turgor pressure in S. aureus cells. Notably, both substances strongly reduced the release of SitC-containing MVs (Fig. 4H and Fig. S2B). Thus, a high turgor in addition to a PSM-mediated increase in membrane fluidity is essential for membrane vesicle release by S. aureus.

10.1128/mBio.01851-18.2 FIG S2 Influence of turgor pressure on vesicle biogenesis. (A) Growth of USA300 wild type in medium containing the indicated turgor-affecting substances. (B) Lipid amounts in MV isolates recovered from bacteria grown in media containing the indicated turgor-affecting substances. Data in panel A represent means and data in panel B represent means ± SEMs from at least three independent experiments. ns, not significant; *, *P* < 0.05, significant difference versus untreated bacteria as calculated by the unpaired, two-tailed Student *t* tests. Download FIG S2, TIF file, 0.2 MB. Copyright © 2018 Schlatterer et al. 2018 Schlatterer et al. This content is distributed under the terms of the Creative Commons Attribution 4.0 International license.

### High concentrations of PSMs and other surfactants destroy S. aureus membrane vesicles, which promotes the proinflammatory activity of S. aureus lipoproteins.

**FIG 5:**
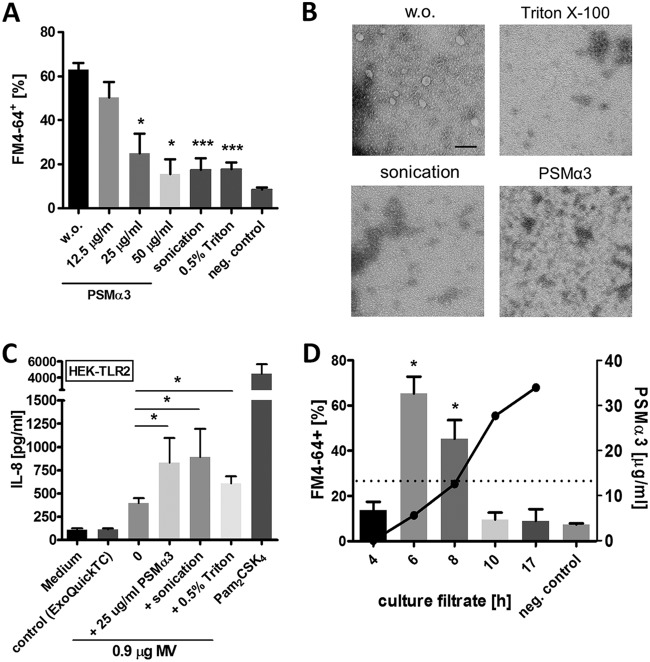
Role of PSMs in vesicle biogenesis and vesicle disruption. (A) Flow cytometric analysis shows disruption of wild-type membrane vesicles through PSMα3 (>12.5 µg/ml), sonication, or 0.5% Triton X-100. (B) TEM of vesicles disrupted by PSMα3, sonication, or Triton X-100 (scale bar, 0.1 µm). (C) Disrupted membrane vesicles show higher activation of TLR2-transfected HEK293 cells. TLR2 agonist Pam_2_CSK_4_ (300 ng/ml) was used as a positive control. (D) Membrane vesicle counts from USA300 wild type at different culture time points analyzed by flow cytometry (bars) and the corresponding PSMα3 concentration (dots) in the culture filtrates measured by HPLC (dashed line, 12.5 µg/ml PSMα3). Data in panels A, C, and D represent means ± SEMs from at least three independent experiments. ns, not significant; *, *P* < 0.05; ***, *P < *0.001, significant difference versus the untreated control (A and C) (w.o. or medium) or negative control (D) as calculated by unpaired (A and D) or paired (C) two-tailed Student’s *t* tests. (B) One representative experiment.

To analyze the stability of lipoprotein- and PSM-containing vesicles, the preparations were treated with the nonionic detergent Triton X-100, with sonication, or with high concentrations of PSMα3. Notably, all treatments led to a decay of FM4-64-positive MV particles, as measured by flow cytometry (Fig. 5A) and as verified by TEM (Fig. 5B). While PSMs mediate the release of membrane vesicles at low concentrations (<12.5 µg/ml), they were found to destroy them at very high concentrations (>12.5 µg/ml). This phenomenon correlates with the present finding that MVs can mostly be isolated from bacterial cultures at between 6 and 8 hours of cultivation (Fig. 5D), when the PSM-controlling quorum-sensing system Agr is most active (23). Under these conditions, the PSMα3 concentration in culture supernatants was below 12.5 µg/ml. After 10 hours of growth, when Agr strongly reduces its activity (23), PSMα3 concentrations reached higher values, and consequently, only small amounts of MVs were found in wild-type culture supernatants (Fig. 5D).

The proinflammatory motif of lipoproteins, the characteristic lipid anchor, is buried in the membrane when lipoproteins are embedded in MVs, which raises the question of how lipoproteins can reach TLR2. Vesicle-disrupting surfactants could increase the availability of lipoproteins for TLR2 binding, but they could also cover the hydrophobic fatty acid chains in a way that would abrogate its biological activity. When MV preparations were treated with vesicle-disrupting concentrations of Triton X-100 or PSMα3 or by sonication, their capacity to stimulate TLR2-transfected HEK293 cells was significantly increased (Fig. 5C and Fig. S3A), indicating that lipoproteins must be released from MVs to exert their maximal stimulating activity and that surfactants do not abrogate but promote the activity of lipoproteins.

10.1128/mBio.01851-18.3 FIG S3 Stimulation of HEK-TLR2 and HL60-FPR2 cells with Triton X-100-disrupted vesicles. (A) HEK-TLR2 stimulation with intact or with 0.5% Triton X-100-disrupted vesicles (0.5% MV isolation) derived from wild-type and Δ*α,β,hld* mutant cells. TLR2 activation was measured by IL-8 cytokine secretion. (B) Flow cytometric analysis of calcium mobilization in FPR2-transfected HL60 cells after incubation with intact or 0.5% Triton X-100-disrupted vesicles (0.005% MV isolation). Data represent means ± SEMs from at least three independent experiments. ns, not significant; *, *P* < 0.05, significant difference versus untreated vesicles (w.o.) as calculated by the paired, two-tailed Student *t* tests. Download FIG S3, TIF file, 0.2 MB. Copyright © 2018 Schlatterer et al. 2018 Schlatterer et al. This content is distributed under the terms of the Creative Commons Attribution 4.0 International license.

## DISCUSSION

Lipoproteins are major bacterial MAMPs with particularly important roles in infections caused by Gram-positive bacteria (2, 3), and they can cause exuberant inflammation and contribute to the severity of diseases or orchestrate host defense in a beneficial, sometimes even anti-inflammatory fashion (24). Bacterial pathogens vary strongly in the amounts of lipoproteins that they release (25), and S. aureus has been found to modulate TLR2 activation, for instance, by controlling lipoprotein release via the quorum-sensing system Agr (4) or by producing the TLR2-inhibitory protein SSL3 (15). Many aspects of the pathway, from the bacterial release of lipoproteins to their activation of TLR2, have remained unclear. Lipoproteins have difficult physicochemical properties because the hydrophobic fatty acids limit their solubility. Interestingly, the same is true for PSM peptides and their capacity to stimulate FPR2 (8). How lipoproteins are released from bacterial membranes has remained largely unclear, considering that the extraction of fatty acid chains from the cytoplasmic membrane is regarded as an energy-dependent process. PSMs have been found to promote the release of lipoproteins, but the mechanism has remained unknown.

We demonstrate here that lipoproteins are not released as individual molecules but as components of larger MVs. Such vesicles were released only under hypotonic conditions, indicating that strong turgor provides the energy for the constriction of MVs. Factors that impact on peptidoglycan cross-linking, such as autolysins or antibiotics, have been found to influence the amount and size of membrane vesicles probably because they govern the capacity of MVs to penetrate the cell wall (26). The strong curvature that the membrane has to undergo during vesicle budding requires a high level of fluidity. It seems that the surfactant-like properties of PSMs impart the necessary level of fluidity to the membrane, which may explain why MVs are released only in the presence of PSMs and why PSMαs with particularly strong amphipathic properties have the highest MV-releasing capacity. It remains to be analyzed how other bacterial, environmental, or host-derived detergents may affect the release of lipoprotein-containing MVs. It should be noted that the membrane-active antibiotic daptomycin has been shown to promote the release of phospholipids from S. aureus (17). The release of TLR2 agonists by skin-associated bacteria is thought to contribute to local inflammation, in particular in chronic disorders such as atopic dermatitis (27). Along this line, S. aureus-derived MVs have been shown to cause strong skin inflammation in a mouse model (28). It will be important to analyze how the components of skin lotions and soaps may facilitate lipoprotein release. Extensive use of skin detergents is known to augment skin inflammation in atopic dermatitis (26), which may in part be due to the mobilization of proinflammatory TLR2 agonists.

Detergent-like molecules such as PSMα3 not only promoted release of vesicles from the S. aureus cytoplasmic membrane but also induced their disintegration at high concentrations. Notably, vesicle disintegration was accompanied by an increased capacity of lipoproteins to activate TLR2, suggesting that solubilized lipoproteins can reach the ligand binding pocket of TLR2 more easily than membrane-embedded lipoproteins. The activating motif of lipoproteins is the lipid anchor with its fatty acid chains, which is usually not accessible to TLR2 as long as it is attached to the membrane. Surfactant-like molecules may thus be essential for effective TLR2 activation. Bacterial lipopolysaccharide (LPS), the agonist of TLR4, also needs to be released from membranes to exert its proinflammatory activity (29). Accessory host proteins, such as LPS-binding protein (LBP) and CD14, are thought to facilitate the release of LPS from vesicles and promote their integration into TLR4 (29, 30). Of note, LBP, CD14, and CD36 have also been implicated in TLR2 activation (30–32), which raises the possibility that these or further, yet-to-be-identified host proteins can contribute to the membrane vesicle extraction of lipoproteins. Some reports have also described the fusion of bacterial MVs with host cells (28), which could transfer lipoproteins into host cytoplasmic membranes and allow them to reach TLR2 by lateral diffusion.

The release of MVs by S. aureus and other bacteria has been reported by several laboratories in the past, and some described TLR2-dependent proinflammatory properties of such structures, which is in agreement with our findings (16, 28). Different methods have been described for MV isolation, including precipitation by high-speed centrifugation (33), density gradient centrifugation, and size exclusion centrifugation (18). We compared density gradient centrifugation with a new, particularly convenient method, based on the MV isolation reagent ExoQuickTC, which has been developed for preparation of eukaryotic exosomes (34). We demonstrate that ExoQuickTC preparations yield very similar results in several microscopy, flow cytometry, and bioactivity-based assays and lead to even higher MV yields than density gradient centrifugation. Thus, the new technique may strongly facilitate future research on bacterial MVs and on the potential application of MVs for vaccination purposes (35). Some previous studies have attributed cytotoxic properties to S. aureus MVs (16), which may be due to vesicle-associated PSM peptides. Some have also reported the presence of secretory toxins in MVs, such as γ-hemolysin, leucocidin D, and exfoliative toxin C (36), which is unexpected because the content of vesicles should be derived from the bacterial cytoplasm. However, even secretory proteins could remain associated with the membranes of MVs. Careful evaluation of the purity and absence of cell debris will be important for future studies on the molecular properties of bacterial MVs.

Our findings suggest that PSMs may use two different strategies to exit bacterial cells—the previously described Pmt ABC transporter, probably taking PSMs up from the membrane and excreting them as free molecules (37), and the release of PSM-containing MVs. Our study also underscores the crucial roles of PSMs in the release of membrane-embedded and cytoplasmic proteins ranging from the mobilization of protein-containing MVs to the disintegration of vesicles at high concentrations, which leads to the release of free lipoproteins and cytoplasmic proteins. The release of cytoplasmic proteins by Gram-positive bacteria, some of which have moonlighting activities when they are extracellular, has been documented in several studies (18). The surfactant-promoted release of membrane vesicles may represent the major pathway for their release.

## MATERIALS AND METHODS

### Bacterial cultivation and preparation of culture filtrates.

Bacterial strains (see Table S1 in the supplemental material) were maintained on sheep blood tryptic soy agar plates. Hemolysis on blood agar plates and RNAIII expression were monitored to confirm functional Agr systems and toxin production in S. aureus USA300. All bacteria were grown in tryptic soy broth (TSB) or in TSB without glucose supplemented with 0.5% xylose (S. aureus USA300 pTX SitC-His strains). Bacterial cultures were supplemented with the appropriate antibiotics and grown in flasks on a 37°C shaker, and culture supernatants were obtained by centrifugation of 6-h or 10-h cultures by filtration through 0.2-μm-pore-size filters.

10.1128/mBio.01851-18.4 TABLE S1 Bacterial strains and deletion mutants used in this study. Download Table S1, DOCX file, 0.01 MB. Copyright © 2018 Schlatterer et al. 2018 Schlatterer et al. This content is distributed under the terms of the Creative Commons Attribution 4.0 International license.

### Vesicle isolation from bacterial culture filtrates.

To obtain size-separated culture supernatants, sterile-filtered culture supernatants from late exponential growth phase (6 h) of S. aureus USA300 were transferred onto 100-kDa centrifugal concentrator cartridges (Vivaspin 20; Sartorius) and centrifuged at 3,000 × *g*. The >100-kDa fraction was resuspended in 1 ml PBS or TSB. For vesicle isolation with the ExoQuickTC kit (EQPL10TC; System Bioscience), the over-100-kDa culture filtrate fractions were incubated overnight at 4°C with ExoQuickTC at a ratio of 5:1. Vesicles were then pelleted by centrifugation at 1.500 rpm for 30 min and resuspended in 1 ml fresh PBS (Fig. 2A).

For vesicle isolation by OptiPrep (D1556, Sigma-Aldrich) density gradient ultracentrifugation, the >100-kDa fraction was resuspended in PBS, and the vesicles were pelleted by ultracentrifugation (3 h, 100,000 × *g*, 4°C) using a T29 rotor (ThermoFisher). The pellet was then resuspended in 40% OptiPrep and overlaid with OptiPrep dilutions ranging from 35% to 10%. The gradient was centrifuged in a SW40 rotor (Beckmann) for 16 h at 139,000 × *g*. The different density fractions were then collected, and the fractions (35% to 20%) that showed a similar protein pattern by silver staining (Fig. S1A) were pooled. These pooled fractions were concentrated using a 100-kDa concentrator cartridge and further referred to as MV isolates (Fig. 2A).

### Lipid and protein quantification.

The fluorescent membrane dye FM4-64 (Life Technologies) was used to quantify the lipid amount in culture filtrates, size-separated culture filtrate fractions, or vesicle isolates from S. aureus USA300 wild type and USA300 Δ*α,β,hld*. The different fractions were stained at 37°C for 5 to 10 min with FM4-64 at a final concentration of 5 μg/ml, and lipid positivity was detected using the fluorescence microplate reader CLARIOStar (BMG Labtech). Determination of the protein amount was performed using a Bradford assay according to the manufacturer’s manual (Bio-Rad protein assay kit).

Silver staining was used to detect smaller protein amounts in MVs isolated by OptiPrep ultracentrifugation. SDS-PAGE was performed as described below, and the total MV isolate was applied to an SDS gel. A silver staining kit (Bio-Rad) was used according to the manufacturer’s instructions.

### Negative staining for transmission electron microscopy (TEM).

MV isolates were gained as described above. ExoQuickTC pellets were resuspended in 20 µl PBS, and pooled fractions from OptiPrep were concentrated to a final volume of 50 µl. All samples were fixed with 1:1 Karnovsky’s fixative. Suspensions were placed directly onto a glow-discharged electron microscopy (EM) grid. After adsorption, the grids were washed in double-distilled water and negatively stained with 1% uranyl acetate. The grids were examined using a Zeiss Libra 120 transmission electron microscope (Carl Zeiss, Oberkochen, Germany) operated at 120 kV. Original magnification was 1:25,000.

Bacterial cultures were grown for 6 h, diluted 1:1,000, and centrifuged at 4,700 × *g* for 10 min. Bacteria were fixed with Karnovsky’s fixative for 24 h at 4°C. Postfixation bacteria were placed in 1.0% osmium tetroxide containing 1.5% K-ferrocyanide in 0.1 M cacodylate buffer for 2 h. Blocks were embedded in glycide ether and cut using an ultramicrotome (Ultracut; Reichert, Vienna, Austria). Ultrathin sections (30 nm) were mounted on copper grids and analyzed using a Zeiss Libra 120 transmission electron microscope (Carl Zeiss, Oberkochen, Germany) operating at 120 kV.

### Dynamic light scattering for size analysis.

Size determination of isolated vesicles was performed using dynamic light scattering analysis with a Zetasizer Nano ZS (Malvern Instruments) according to the manufacturer’s instructions.

### SitC, PSMα3, and cytoplasmic protein detection.

To induce SitC expression in S. aureus USA300 pTX SitC-His, bacteria were cultivated in TSB without glucose containing 0.5% xylose. Bacterial cultures were adjusted to densities of OD_600_ of 0.1 and cultivated for appropriate times in flasks under agitation at 37°C. For turgor modulation, bacterial cultures were supplemented with the indicated percentage of sucrose or arabinose. MVs were obtained by centrifugation, as described above, and used for detection of SitC-His or PSMα3 by immunoblotting. A volume corresponding to 50 μg of total protein was concentrated with 10 μl Strataclean resin beads (Agilent Technologies) and loaded onto Mini-Protean TGX precast protein gels (Bio-Rad). SitC detection was performed as described recently (4) using mouse anti-5His-IgG from Qiagen (0.2-mg/ml stock solution diluted 1:1,000). Goat anti-mouse-IgG IRDye680 or IRDye800 purchased from Li-Cor (0.2-mg/ml stock solution diluted 1:10,000 in Tris-buffered saline [TBST] with 2% BSA) was used as secondary antibody. PSMα3 probes were prepared as described for SitC-His, but the probes were loaded on 10% to 20% Tris-glycine minigels (Novex) and detected using anti-PSMα3 serum (isolated by M. Otto) and mouse anti-rabbit-IgG IRDye800 from Li-Cor (0.2-mg ml^−1^ stock solution diluted 1:10,000). Samples used for the detection of the cytoplasmic protein GAPDH in ExoQuickTC-isolated vesicles were prepared as described for the SitC sample preparation. GAPDH was detected using a specific primary antibody (α-GAPDH [38]) and a secondary anti-rabbit-IgG IRDye680 antibody. All bands on the membranes were visualized by Li-Cor Reader.

### Quantitative label-free proteomics.

Three biological replicates of ExoQuickTC-isolated MVs from USA300 wild type or PSM mutant were analyzed. Volumes corresponding to similar protein amounts in all MV isolates were measured by Bradford assay and used for protein precipitation with 10% ice-cold trichloroacetic acid (TCA) overnight at 4°C. After centrifugation at 13,200 rpm at 4°C for 15 min, the supernatants were discarded, and the precipitated proteins were air-dried. Nano-liquid chromatography–tandem mass spectrometry analysis was performed as described recently (4). Briefly, dried proteins were dissolved in a buffer containing 6 M urea, 2 M thiourea, and 10 mM Tris at pH 8.0 and digested in solution with trypsin. Peptide mixtures from the samples were separated on an EasyLC nano-high-performance liquid chromatograph (Proxeon Biosystems) coupled to a linear trap quadrupole (LTQ) Orbitrap Elite mass spectrometer (Thermo Fisher Scientific). Acquired mass spectrometry spectra were processed as described previously (4). Differences of single proteins between the wild type and PSM mutant are listed in Data Set S1.

### Stimulation of HEK-TLR2 cells.

HEK293 cells stably transfected with the human TLR2 genes were purchased from Invivogen. HEK-TLR2 cells were cultivated in 75-cm^2^ culture flasks using 20 ml of growth medium (Dulbecco’s modified Eagle’s medium [DMEM], 10% fetal calf serum [FCS], 20 mM l-glutamine, 100 μg/ml Normocin, and 10 μg/ml blasticidin). Cells were stimulated as described previously (4).

### Calcium mobilization in HL60-FPR2 cells.

HL60 cells stably transfected with human FPR2/ALX have been recently described (5). These cells were grown in RPMI medium (Biochrom) supplemented with 10% FCS (Sigma-Aldrich), 20 mM HEPES (Biochrom), penicillin (100 units/ml), streptomycin (100 µg/ml) (Gibco), 1× GlutaMAX (Gibco), and G418 (Biochrom) at a final concentration of 1 mg/ml. Calcium fluxes were analyzed by stimulating cells loaded with Fluo-3-AM (Molecular Probes), and the fluorescence was monitored with a FACSCalibur flow cytometer (Becton, Dickinson), as recently described (39).

### FACS analysis.

Membrane vesicle isolates from late-exponential-growth-phase (6-h) cultures were stained with 5 μg/ml FM4-64 (Life Technologies) for 20 min at 37°C and analyzed with a BD Bioscience LSRFortessa. SitC-His was detected in vesicle isolates from USA300 *Δspa* using a His-PE antibody (BioLegend), and the staining was controlled using the corresponding PE isotype control (BioLegend). For analysis of cytoplasmic GFP, vesicles were isolated from USA300 containing the pTX143-S3 GFP plasmid. The correlation of FM4-64-positive events with total events was used to calculate the vesicle concentrations in the samples. FlowJo V10 was used for the data analysis.

### Membrane fluidity assay.

Overnight cultures of USA300 wild type and USA300 Δ*α,β,hld* were adjusted to an OD_600_ of 0.2 in Iscove’s modified Dulbecco’s medium (IMDM; Gibco) and stained for 20 min at 37°C with fluorescent lipid reagent supplied in the membrane fluidity kit (ab189819; Abcam). The stained bacteria were centrifuged for 10 min at 5,000 × *g* and resuspended in PBS with 0.2% glucose. Next, the bacteria were incubated with indicated stimuli for 10 min. Formylated PSM peptides (PSMα1, PSMα2, PSMα3, and δ-toxin [*hld*]) with the recently published sequences (7) were kindly provided by Stefan Stevanović (Department of Immunology, University of Tübingen, Germany). Membrane fluidity was analyzed in the fluoreader CLARIOstar (BMG Labtech) according to the manual instructions. A ratio between the emission maxima of the excimer (470 nm) and the monomer (400 nm) was calculated, which is equivalent to the relative membrane fluidity.

### HPLC analysis of PSM peptides.

The S. aureus strain USA300 wild type was grown in TSB at 37°C. Samples were collected at different time points and centrifuged for 10 min at 4,700 × *g* and 4°C. Supernatants were collected by sterile filtration through 0.2-µm filters and concentrated 5 times using a SpeedVac vacuum concentrator. PSM peptides were separated from the supernatant by reversed-phase chromatography using an XBridge C_8_ 5-µm, 4.6 - by 150-mm column (Waters). A linear gradient from 0.1% TFA (buffer A) in water to acetonitrile containing 0.08% TFA (buffer B) for 15 min with an additional 5 min of 100% buffer B at a flow rate of 1 ml/min was used, and a 50-µl sample volume was injected. Peaks were detected at 210 nm. A PSMα3 standard curve was used to calculate the PSMα3 amounts.

### Statistics.

Statistical analysis was performed using GraphPad Prism 5.0. The unpaired two-tailed Student *t* test was used to compare two groups unless otherwise noted. Data represent the mean and SEM from at least three independent experiments unless stated otherwise.
