# Supplementary figures and images for: The Mechanism behind Bacterial Lipoprotein Release: Phenol-Soluble Modulins Mediate Toll-Like Receptor 2 Activation via Extracellular Vesicle Release from Staphylococcus aureus
Source: mBio. 2018 Nov 20;9(6):e01851-18. doi: 10.1128/mBio.01851-18 (PMC6247081; doi:10.1128/mBio.01851-18)

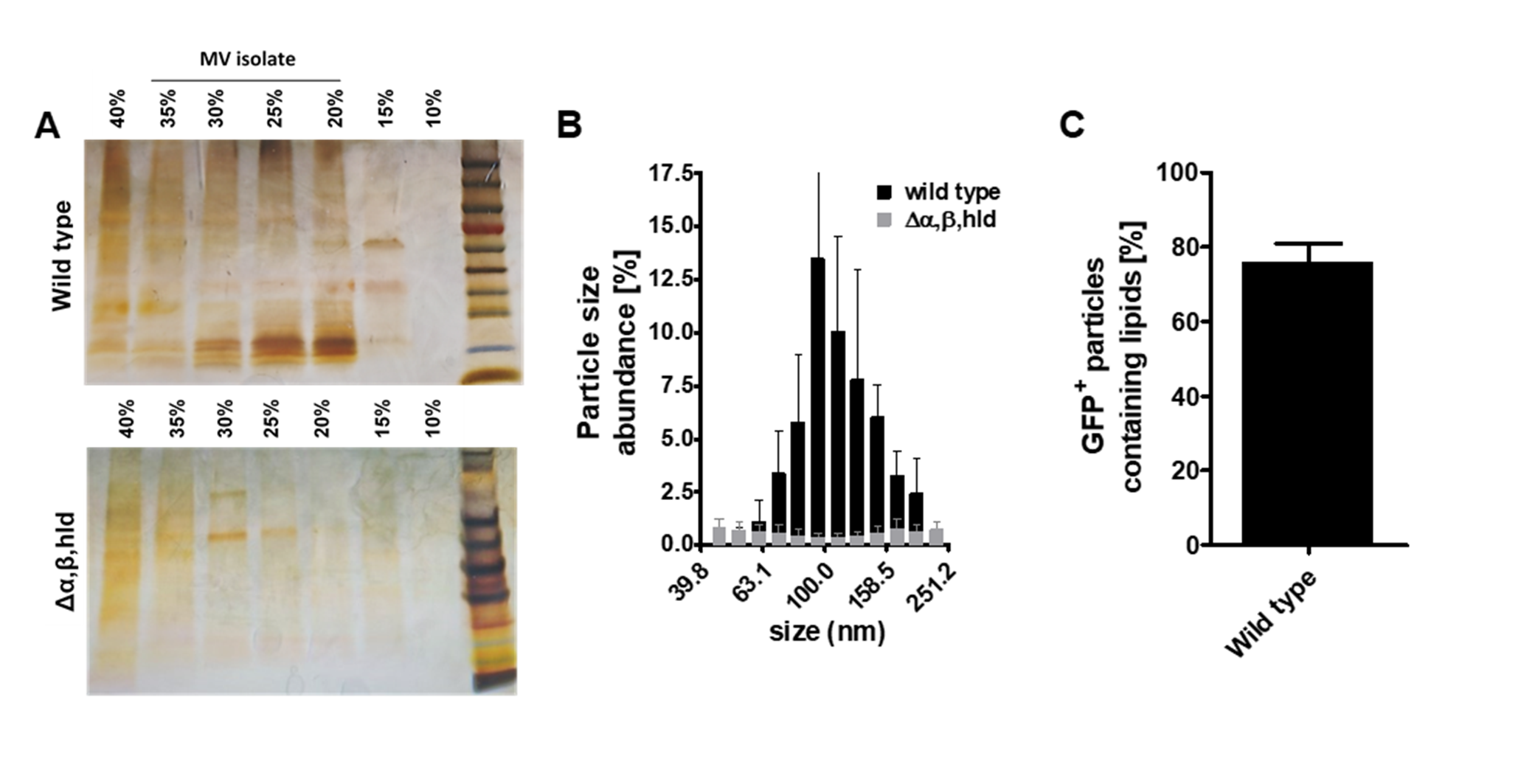

Supplement: FIG S1 [file mbo006184185sf1.tif]

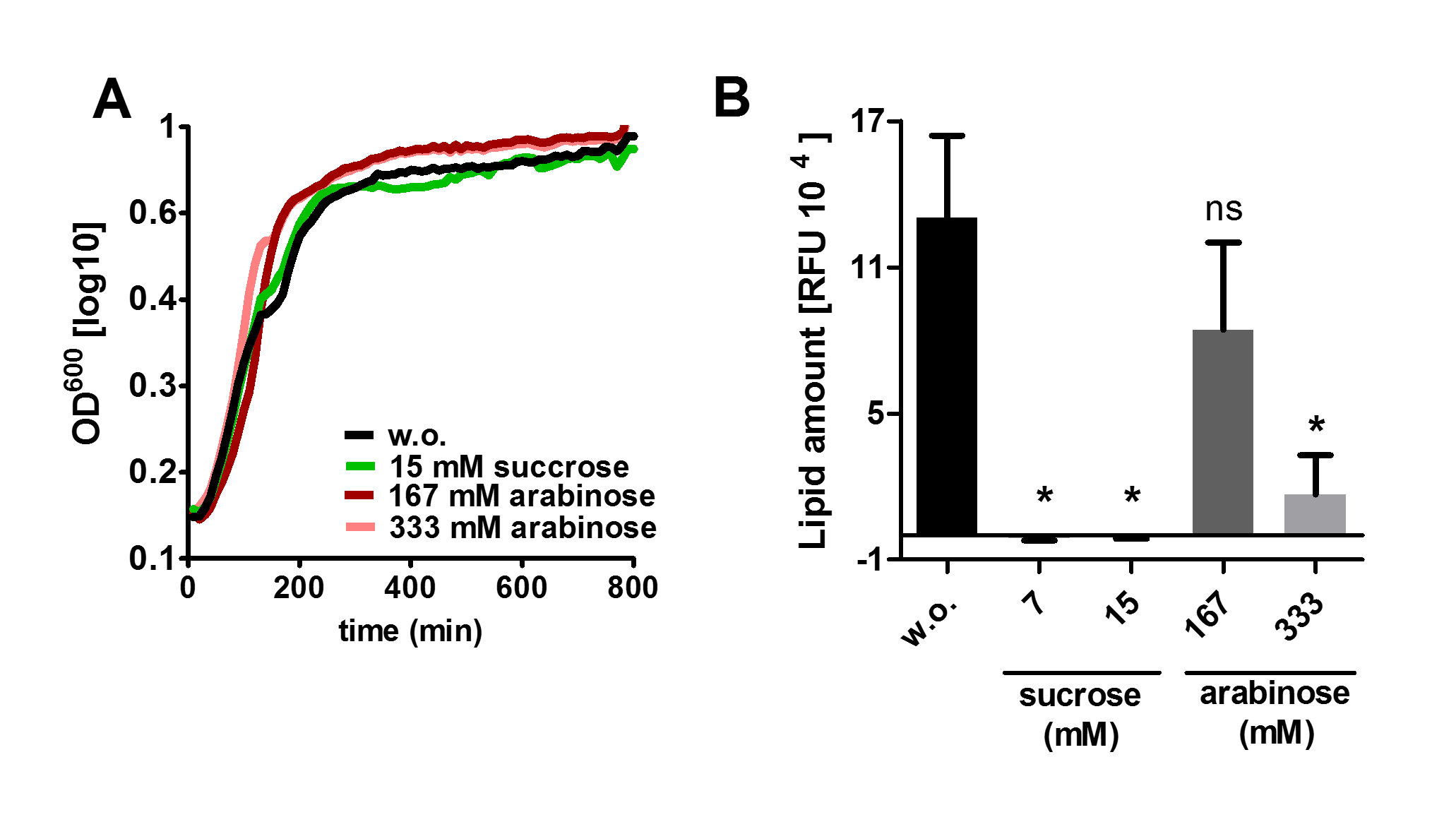

Supplement: FIG S2 [file mbo006184185sf2.tif]

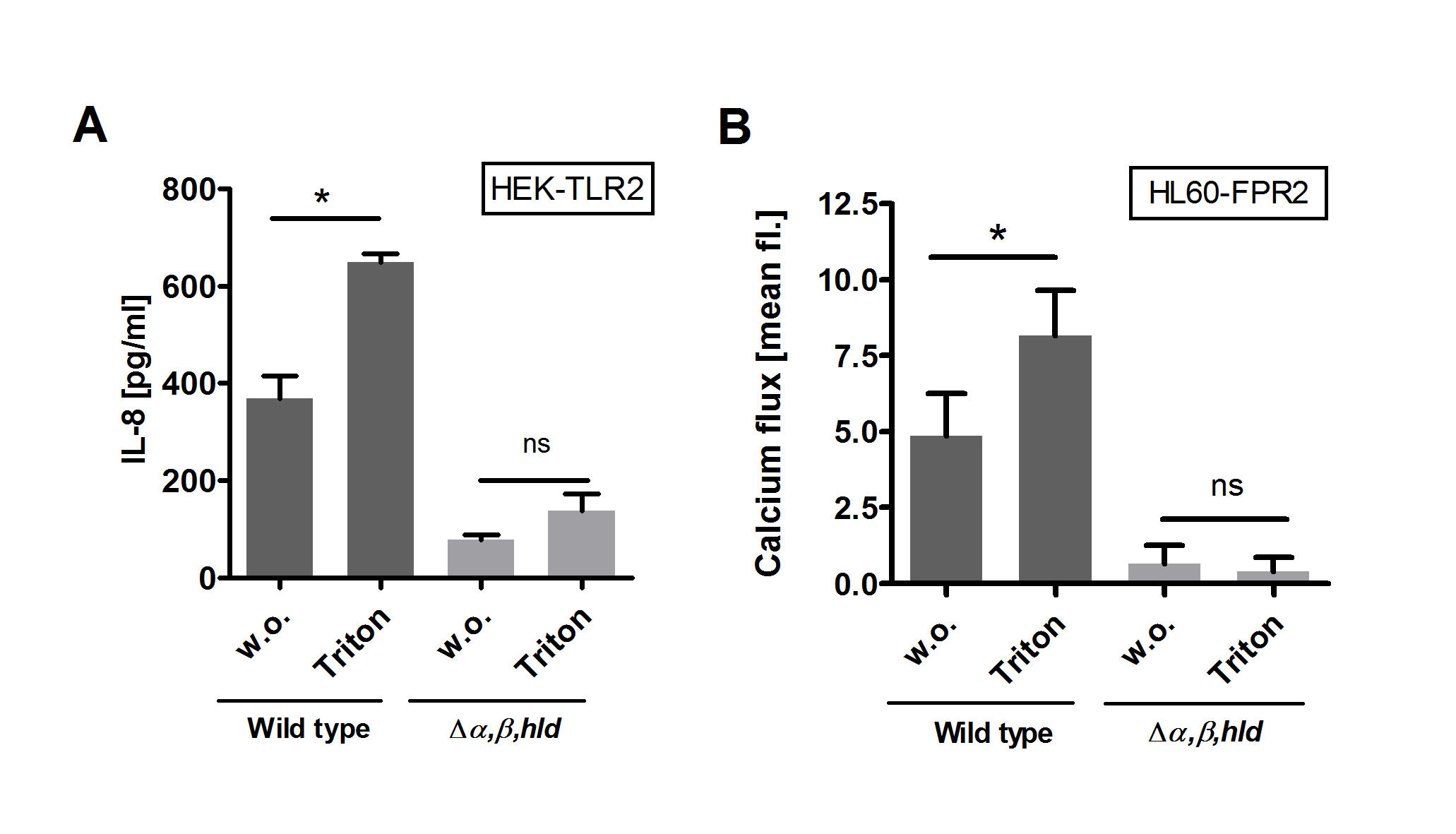

Supplement: FIG S3 [file mbo006184185sf3.tif]
