# Supplementary material for: The Mechanism behind Bacterial Lipoprotein Release: Phenol-Soluble Modulins Mediate Toll-Like Receptor 2 Activation via Extracellular Vesicle Release from Staphylococcus aureus
Source: mBio. 2018 Nov 20;9(6):e01851-18. doi: 10.1128/mBio.01851-18 (PMC6247081; doi:10.1128/mBio.01851-18)
Supplement: TABLE S1 [file mbo006184185st1.docx]

**Supplementary Table 1: Bacterial strains and deletion mutants used in this study.**

| **Species** | **Strain** | **Genotype** | **Reference** |
| --- | --- | --- | --- |
| *Staphylococcus aureus* | USA300 LAC | Wild type | Wang, R., et al. 2007 |
| *Staphylococcus aureus* | USA300 LAC | *Δα,β,hld* | Joo, H. S., et al. 2011 |
| *Staphylococcus aureus* | USA300 LAC | *Δα1-4* | Wang, R., et al. 2007 |
| *Staphylococcus aureus* | USA300 LAC | *Δagr* | Wang, R., et al. 2007 |
| *Staphylococcus aureus* | USA300 LAC | *Δspa* | Ebner, P., et.al. 2016 |
| *Staphylococcus aureus* | USA300 LAC | *Δlgt* | Hanzelmann, D., et.al. 2016 |
| *Staphylococcus aureus* | Sa113 (MSSA) | Wild type | Stoll, H. et al. |
| **Plasmid** | **Purpose** | **Resistance** | **Reference** |
| pTX30SitC-His | Xylose induced SitC-His expression | tetracyclin 12,5 μg/ml | Stoll, H., et al. 2005 |
| pTX143-S3-GFP | Cytoplasmic GFP expression | tetracyclin 12,5 μg/ml | - |
| ptXΔ16 | Empty control vector | tetracyclin 12,5 μg/ml | Wang, R., et al. 2007 |
| pTXΔα1-4 | Constitutiv expression of psmα1-4 | tetracyclin 12,5 μg/ml | Wang, R., et al. 2007 |
